# The Effect of the Tumor Microenvironment and Tumor-Derived Metabolites on Dendritic Cell Function

**DOI:** 10.7150/jca.38785

**Published:** 2020-01-01

**Authors:** Jun-Ho Lee, So-Yeon Choi, Nam-Chul Jung, Jie-Young Song, Han Geuk Seo, Hyun Soo Lee, Dae-Seog Lim

**Affiliations:** 1 Department of Biotechnology, CHA University, 335 Pangyo-ro, Bundang-gu, Seongnam, Gyeonggi-do 13488, Republic of Korea.; 2 Pharos Vaccine Inc., 545 Dunchon-daero, Jungwon-gu, Seongnam, Gyeonggi-do 13215, Republic of Korea.; 3 Department of Radiation Cancer Sciences, Korea Institute of Radiological and Medical Sciences, 75 Nowon-ro, Nowon-gu, Seoul 01812, Republic of Korea.; 4 Department of Food Science and Biotechnology of Animal Products, Sanghuh College of Life Sciences, Konkuk University, 120 Neungdong-ro, Gwangjin-gu, Seoul 05029, Republic of Korea.

**Keywords:** dendritic cells, tumor microenvironment, exogenous factor, metabolite.

## Abstract

Dendritic cells (DCs) have a critical effect on the outcome of adaptive immune responses against growing tumors. Recent studies on the metabolism on DCs provide new insights on the functioning of these critical controllers of innate and adaptive immunity. DCs within the tumor microenvironment (TME) often exist in an inactive state, which is thought to limit the adaptive immune response elicited by the growing tumor. Tumor-derived factors in the TME are known to suppress DC activation and result in functional alterations in DC phenotype. We are now beginning to appreciate that many of these factors can also induce changes in immune cell metabolism. In this review, we discuss the functional alternation of DC phenotype by tumor metabolites.

## Introduction

Dendritic cells (DCs) are the most potent professional antigen-presenting cells (APCs) and have a central role in maintaining immune homeostasis 1,2. DCs are not only important for induction of primary immune responses, but may also have a pivotal role in priming T cell-mediated immune responses 3. Active immunotherapy with dendritic cells can be used to induce anti-tumor immune responses and therefore offers an attractive alternative to conventional cancer treatments 4,5. The recent development of methods for large-scale *ex vivo* production of DCs from peripheral blood monocytes has contributed to their growing use in cancer vaccination trials 6. The primary goal of DC-based immunotherapy is to induce an antigen-specific immune response 7,8. The effector arm of anti-tumor immunity is comprised of CD4^+^ and CD8^+^ T cells, which can only recognize antigen when it is presented by APCs 8. Therefore, DCs are currently considered attractive therapeutic targets for use in combination with immune checkpoint inhibitors 9. In the case of cancer vaccines, the DCs themselves are administered as the therapy. Preparation of a DC vaccine involves the generation of DCs from CD34^+^ precursor cells or monocytes, loading the DCs with specific tumor antigens, and, finally, administration of the cells to the patient. Upon administration, the DCs must migrate to the secondary lymphoid organs and induce an antigen-specific immune response. DC-based cancer vaccines have been tested in clinical trials of patients with melanoma, myeloma, prostate cancer, renal cancer, breast cancer, ovarian cancer, and gastrointestinal cancer. Although there is evidence that DC vaccines can induce anti-tumor immune responses in some patients, clinical results remain poor. Multiple clinical studies have indicated that DCs within tumors are scarce and functionally defective 10,11. Although objective clinical responses have been observed, results so far have been somewhat disappointing compared with those obtained *in vitro* and in animal models. There may be multiple reasons for this lack of efficacy, including a tendency for early-phase clinical trials to enroll patients with advanced, metastatic disease and associated comorbidities, including immune suppression, which has only recently been recognized as a major barrier to the efficacy of tumor vaccines in general. Suboptimal maturation and immunogenicity of DCs within early vaccine formulations and the lack of definition of immunogenic neoantigens may also have contributed to the limited clinical efficacy of these strategies 12. These relative failures must be re-interpreted in light of current data, particularly those concerning DC maturation status 13. Using DCs that are not fully matured results in ineffective vaccination against tumor antigens and may even promote immune tolerance, suggesting that vaccines should incorporate signals to achieve full maturation and activation of DCs prior to vaccine administration 14-16. This finding underscores the importance of the DC generation method to the success of a vaccination strategy. DC generation protocols mainly differ in their methods of DC maturation and in their serum components.

Although lipopolysaccharide (LPS) is a commonly used maturation factor in mouse studies, interleukin (IL)-1β, IL-6, tumor necrosis factor (TNF)-α, and prostaglandin E2 (PGE_2_) are commonly used in the maturation of monocyte-derived DCs (mo-DCs) for clinical studies. The maturation stimulus is a major factor that determines the success or failure of DC therapy. TLR agonists [the TLR-4 agonist lipopolysaccharide (LPS), the TLR-3 agonist poly (I:C), and the TLR-7/8 agonist resiquimod], cytokines [TNF-α, IL-1 β, IL-6, interferon (IFN)-α, and IFN-γ)], costimulatory receptor ligands (CD40L), and PGE_2_ have all been used either alone or in various cocktails to mature and program DCs 17. The desired maturation outcome is to induce high expression of MHC molecules, costimulatory molecules such as CD80, CD86, and CD40, and chemokines such as CCR7, as well as the secretion of Th1 cytokines such as IFN-γ, so as to polarize DCs towards Th1 activation 18.

Recently, the generation of DC with different serum components was also reported. Comparative analyses were conducted concerning the impact of various commercially available media and protein supplements on DC generation, maturation, and functional activity 13. In addition, the secretion of immunosuppressive factors by cancer cells has been implicated in the control of DC maturation and function 10,19. Areas of hypoxia and necrosis are common in tumors, which limits the availability of nutrients for both tumor cells and immune cells. However, tumor cells undergo metabolic reprogramming (glycolysis) even in the presence of oxygen, which allows them to sustain their own growth and proliferation, depriving immune cells of nutrients essential for their function and survival. The secretion and accumulation of tumor metabolites, termed oncometabolites, within the tumor microenvironment can also suppress immune cell function [20.21]. The conditioning of immune responses by metabolic challenges within the tumor microenvironment is increasingly becoming studied as a mechanism of tumor immune escape. Here, we discuss the current thinking on the involvement of these tumor metabolites and their effects on the generation of functional mo-DC.

## The effects of protein components and tumor metabolites on DC generation *in vitro*

Several reports analyzed the effects of the culture medium and protein supplements on DC yield, viability, maturation, and function. Myeloid cells are more sensitive to supplementation used in culture media than other immune cells. Indeed, various protein supplements significantly altered the maturation status of DC generated *in vitro* 12. Fetal bovine serum (FBS) may contain xenogeneic proteins as well as pathogenic viruses or prions that deactivate endogenous pancreatic enzymes 13,22. Additionally, immunologic complications may occur, since FBS contains proteins that can induce immune responses. Instead, human serum albumin (HSA) and human serum or plasma are commonly used in the generation of mo-DCs, especially for clinical application. HSA, naturally present in the human body, was evaluated as a potential substitute for FBS in DC cultures 23. Furthermore, as HSA can be purified under Good Manufacturing Practice (GMP) conditions, culture protocols using HSA can be easily standardized. Moreover, the concentration of HSA that is used to generate mo-DCs is on the same order of magnitude as the protein concentration in serum 13. HSA therefore acts as a kind of non-antigen culture substrate and provides the “cleanest” culture microenvironment, but it lacks the natural growth factors found in serum 24, which enhance DC maturation. Indeed, culture in media supplemented with autologous serum or plasma, which contains potential DC growth factors such as ecto- or exo-enzymes and metabolites, may be a good choice for DC generation 24-26. Of course, the collection of autologous serum or plasma is suitable for healthy volunteers, but not patients. Furthermore, active tumor-derived factors in patients' serum could impair the differentiation of monocytes into DCs *in vitro* 24,27-29. It is widely known that many cytokines and immunosuppressive factors detected in the sera of cancer patients can inhibit various stages of DC generation and maturation 30. Onishi et al. showed that monocyte-derived DCs from cancer patients had reduced antigen-presenting capacity compared with DCs generated from healthy volunteers 29. This further supports the beneficial influence of allogenic serum on DC generation.

## Tumor microenvironment-derived factors influence DC maturation and function

The tumor microenvironment (TME) alters the metabolism of tumor-associated immune cells, facilitating tumor cell escape and immune detection. Multiple TME-generated factors contribute to suppressing DC function by inhibiting DC recruitment, activation, antigen presentation, and Th1 polarization 18. A study by Herber et al. in 2009 first reported that high lipid accumulation could render CD11c^+^ CD8^+^ DCs and classical tumor-associated DCs defective in antigen presentation and T cell activation in both murine cancer models and cancer patients 31. Similarly, Cubillos-Ruiz et al. revealed that an inhospitable TME can cause DCs to accumulate endoplasmic reticulum (ER) stress in the form of reactive oxygen species and lipid peroxidation, resulting in abnormal activation of the unfolded protein response (UPR). Aberrant UPR activation in turn induces expression of the transcription factor X-box binding protein (XBP)-1 and consequent inhibition of antigen processing and presentation by DCs 32,33. Intriguingly, treatment with antioxidants and inhibition of XBP-1 expression by using nanoparticles successfully ameliorated ER stress in DCs, recovering their potential to activate T cells and resulting in tumor suppression 18. In the current review, we will focus on the metabolic signals that coordinate DC activation and maturation, and discuss whether targeting these fundamental cellular processes could be used to enhance adaptive immunity.

It is increasingly recognized that the ability of DCs to become activated and prime adaptive immune responses is associated with profound alterations of the cellular metabolic state. Moreover, evidence is accumulating in the context of airway inflammation that various DC functions such as immune priming and immune polarization may have different metabolic requirements. This metabolic plasticity of DC function in airway remodeling could enhance our current understanding of DC metabolism in cancer therapy. Key exogenous metabolites induce the nutrient-sensitive anabolic and/or catabolic pathways to support DC maturation and function 34. Therefore, the metabolic reprogramming of these cells will depend on the tissue environment, nutrient availability, and disease state *in vivo*. Moreover, activation-induced metabolic shift markedly induces the expression of inducible nitric oxide synthase (iNOS) and nitric oxide (NO) production. This is mediated via phosphoinositide 3-kinase (PI(3)K) signaling through mammalian target of rapamycin complex 1 (mTORC1), which promotes a long-term commitment for aerobic glycolysis and anabolic metabolism in inflammatory DCs.

## TME-associated ecto- or exto-enzymes

**Figure 1:**
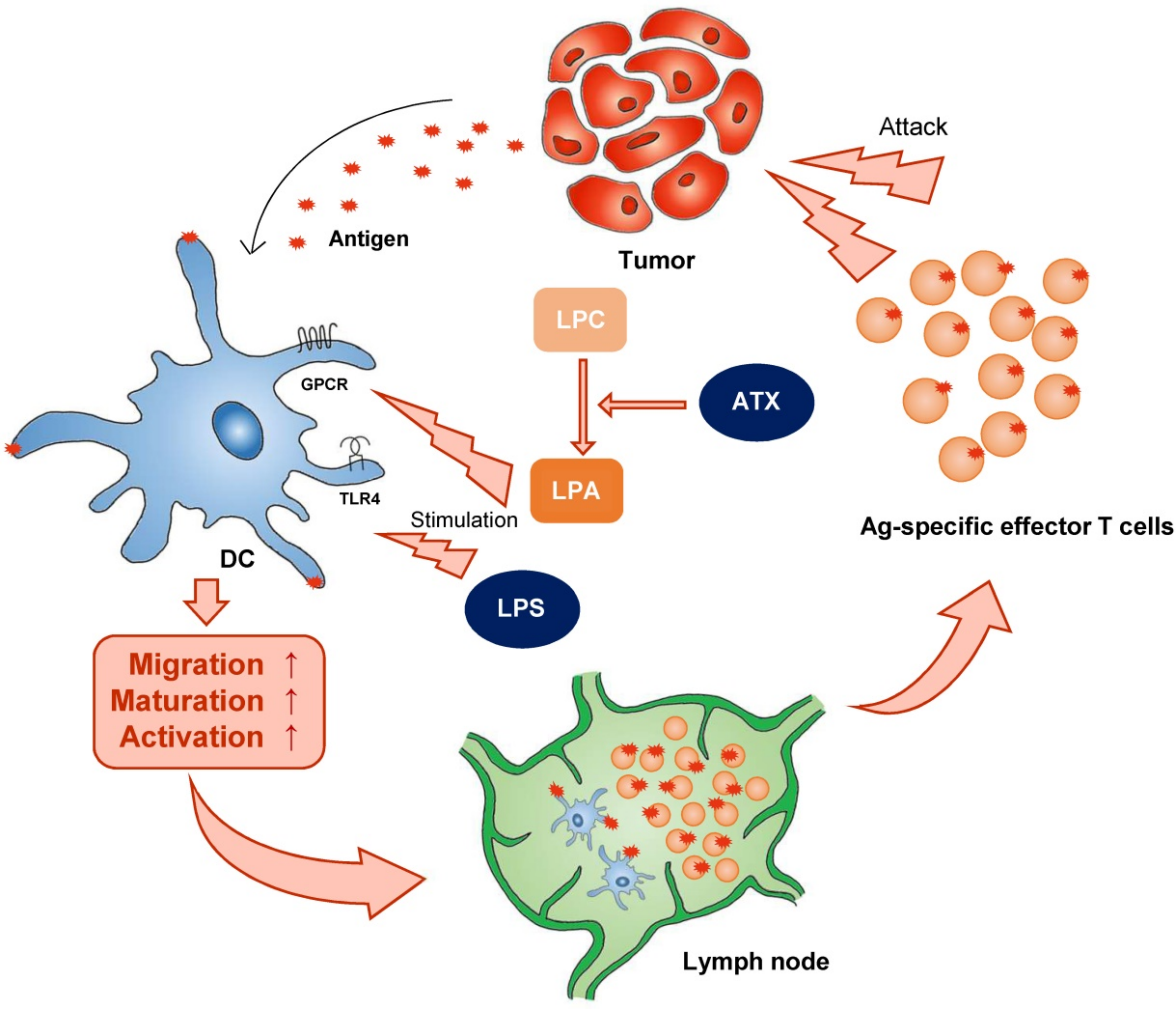
** Classical stimulators induce DC activation.Classical stimulators (e.g. LPA and LPS) influence DC function**. ATX (autotaxin), LPC (lyso -phosphatidylcholine), LPA (lysophosphatidic acid).

There is a growing appreciation that to effectively take up antigen, migrate to the draining lymph node, and present antigen, and therefore to initiate appropriate T cell-mediated immune responses, DCs must undergo the correct metabolic reprogramming. For example, autotaxin (ATX), a secreted lysophopholipase D that is implicated in cancer cell motility, generates extracellular lysophosphatidic acid (LPA) from the precursor lysophosphatidylcholine (LPC). In 1992, ATX was identified as an autocrine factor in A2058 melanoma cells. LPA is a pleiotropic lipid molecule with strong effects on cell growth and migration. LPA induces diverse signaling pathways via six LPA G-protein-coupled receptors (GPCRs), resulting in cytokine production, inflammation, hyperplasia, tumor formation, and metastasis 35. In mice, however, circulating LPA has a half-life of about 3 min. In this aspect, although DC generation media contains LPA, it may be consumed quickly *in vitro*. ATX is associated with various inflammatory diseases including cancer, fibrosis, rheumatoid arthritis, and neural defects. Immature DC derived from LPA3-deficient mice do not migrate to LPA *in vitro*, demonstrating a necessary requirement for this receptor in LPA-directed motility 36. In addition to inducing immature DC migration, LPA can influence DC function (Figure 1). By promoting the mobility of both naive T cells and immature DC, LPA would seem to be well-suited to activate DC:T interactions in lymphoid organs and the initiation of adaptive immune responses.

## Expression of CD39 and CD73 by human tumors

Recently, ecto-nuclase such as CD39 and CD73 were found to be novel immune check-points and potentially new therapeutic targets in solid tumors 37-39. These molecules generate extracellular adenosine at the cancer interface 40. CD39 is the prototype ectonucleoside triphosphate diphosphohydrolase (ENTPD) and was the first of the eight NTPDases described 41,42. Like all NTPDases, CD39 has five highly conserved sequence domains (the apyrase conserved regions). CD39 is anchored to the cell membrane via two transmembrane domains that are essential for maintaining its catalytic activity and substrate specificity 43. CD39 undergoes functional modifications, including limited proteolysis and glycosylation, the latter of which confers catalytic activity to CD39 44. CD73 also exists as a soluble form that is generated by shedding of the glycosylphosphatidylinositol (GPI) anchor and has similar activity as the membrane-bound form 45. Functional CD73 is composed of a non-covalently linked homodimer stabilized by hydrophobic interactions between adjoining C-terminal domains. The purinergic signaling system has crucial effects on tumor growth, survival, and promotion by influencing not only the tumor itself but also immune responses and the tumor microenvironment.

Expression of CD39 and CD73 on tumor cells has been proposed as a prognostic marker. CD39 was found to be highly expressed in pancreatic cancer and is mainly expressed by the vasculature and various stromal cells. A subsequent study performed in the setting of human rectal carcinoma demonstrated increased CD39 expression in tumors and metastases compared with normal tissue, although it was proposed that expression of both CD39 and CD73 leads to poor prognosis 46-48. CD73 expression and its relation to clinicopathological characteristics have been studied in melanoma, acute lymphocytic leukemia, chronic lymphocytic leukemia, glioblastoma, breast cancer, head and neck cancer, ovarian cancer, endometrial cancer, colorectal cancer, prostate cancer, bladder cancer, gastric cancer, kidney cancer, and pancreatic cancer. These studies all revealed that high levels of CD73 expression in the TME were associated with worse clinical outcome 49-52.

## The tumor interface can influence DC function

In solid tumors, adenosine triphosphate (ATP) is abundantly released into the extracellular environment (its concentration can reach a few hundred micromoles per liter, a concentration more than a thousand times higher than in healthy tissues) 53. This is largely due to cell death in the tumor core and to metabolic or hypoxic stress and pro-inflammatory signals that trigger active release of ATP by connexins and pannexin channels expressed by immune and endothelial cells 54. In the tumor microenvironment, extracellular ATP (eATP) acts as a danger signal involved in the infiltration of innate immune cells and in the activation of anti-tumor immune response via the activation of the P2X and P2Y receptors 55. In support for a crucial role of P2 purinergic receptors in the priming of anti-tumor immune response, ATP release in the TME is accounted to be one of the three hallmarks of the process of immunogenic cell death 56. However, in the TME, eATP is usually degraded into immunosuppressive adenosine via the coordinated enzymatic activity of CD39 and CD73 57. As a consequence, in solid tumors, adenosine levels can reach micromolar concentrations. High levels of adenosine can inhibit anti-tumor immunity through the activation of P1 receptors on immune cells. Furthermore, while high concentrations of ATP have been related to enhanced anti-tumor immunity and immune cell-mediated cytotoxicity of tumor cells, low ATP levels due to immoderate ectonucleotidase activity can support tumor growth. Both solid tumors and leukemias produce the ectoenzymes responsible for conversion of ATP into adenosine (immunosuppressive), resulting in suppression of effector T cells and DCs.

**Figure 2:**
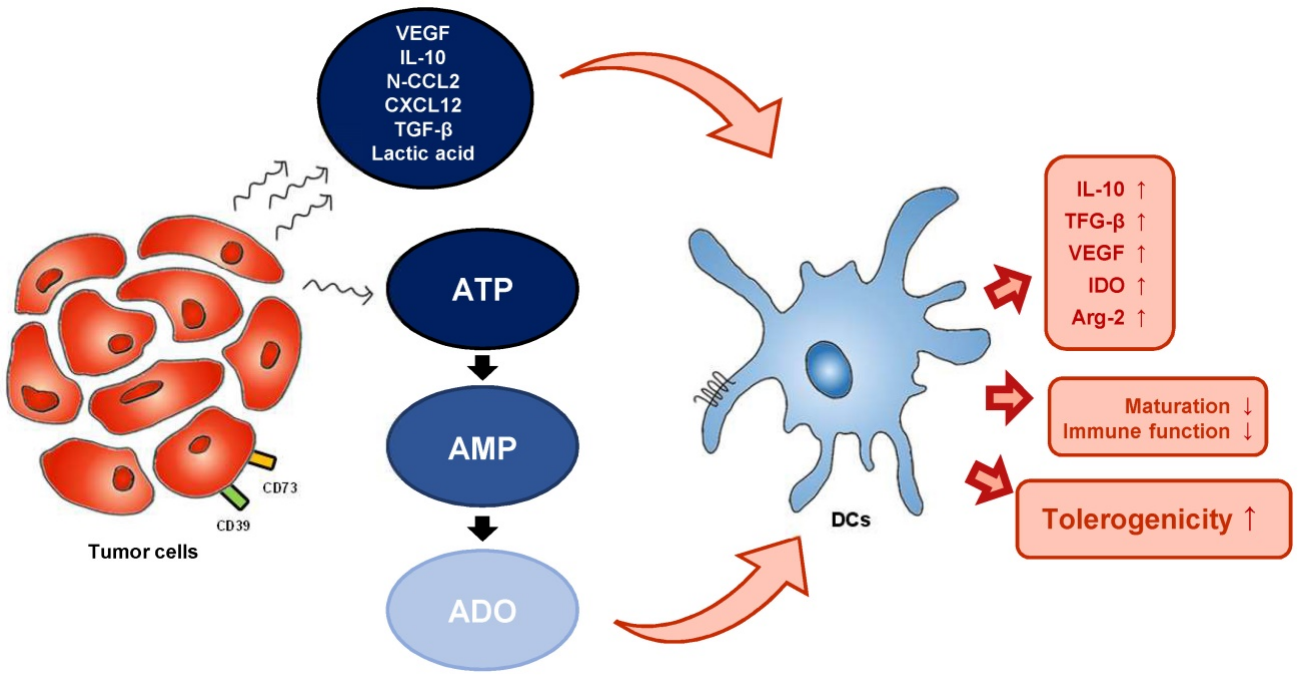
** TME-derived factors can influence DC function. TME and tumor-derived metabolites alter the metabolism of DCs.** TME (tumor microenvironment), VEGF (vascular endothelial growth factor), N-CCL2 (nitrated C-C motif chemokine ligand 2), CXCL12 (C-X-C motif chemokine ligand 12), ATP (adenosine triphosphate), AMP (adenosine monophosphate), ADO (adenosine), IL-10 (interleukin-10), TGF- β (transforming growth factor-β), VEGF (vascular endothelial growth factor), IDO (indoleamine 2.3-dioxygenase), Arg-2 (arginase 2).

The effects of eATP on mo-DCs have been demonstrated in a number of studies. ATP has been found to stimulate human DC maturation, as indicated by high expression of costimulatory molecules such as CD80 58. The expression of these costimulatory molecules and the secretion of IL-12 by ATP-activated mo-DC was mediated by P2Y11R 59. However, exposure of human mo-DCs to eATP gradients inhibited their migratory capacity in a dose-dependent manner, and this effect was also dependent on P2Y11R. Microarray analysis of ATP-stimulated human DCs revealed induction of indoleamine 2.3-dioxygenase (IDO), a potent immunosuppressive factor, suggesting a crucial role for ATP in promoting tolerogenicity 60. CD73-derived adenosine also promotes abnormal differentiation of DCs. Obviously, up-regulation of the A2b receptor on DCs promotes a tolerogenic phenotype characterized by increased production of IL-10, transforming growth factor (TGF)-β, and vascular endothelial growth factor (VEGF), and increased expression of immunosuppressive factors such as IDO and arginase 2. Injection of adenosine or adenosine receptor antagonist-treated DCs into tumor-bearing mice were shown to increase tumor growth, further confirming the impact of adenosine on tumor DCs (Figure 2) 61,62.

## Conclusions

To our knowledge, this is the first review focusing on that tumor-derived metabolites can be greatly associated with negative aspects of DC function in immune system, whereas previous reviews have mainly concentrated on influences on general and broad immune system. Although little research has concentrated on manipulating DC metabolism to induce activation and priming capacity, evidence is accumulating that addresses how metabolic perturbations are linked to the ability of DCs to facilitate immune responses. Taken together, these findings suggest that agents that restore DC metabolic health should be studied, developed, and tested in combination with immune checkpoint blockade. The composition of the culture medium, particularly the presence of metabolites, is as important as other therapeutic products such as cytokines and maturation agents in the function of monocyte-derived DCs, and therefore must be taken into account when establishing standard operating procedures for the generation of DC for experimental use or vaccination. The potential to enhance immune responses to other modalities of immunotherapy makes clinically effective "second-generation" DC vaccination strategies a priority for cancer immunologists.

## Abbreviations

DCs: Dendritic cells
APCs: Antigen-presenting cells
LPS: Lipopolysaccharide
mo-DCs: Monocyte-derived DCs
HAS: Human serum albumin
GMP: Good Manufacturing Practice
TME: Tumor microenvironment
UPR: Unfolded protein response
XBP: X-box binding protein
iNOS: Inducible nitric oxide synthase
NO: Nitric oxide
mTORC1: Mammalian target of rapamycin complex 1
ATX: Autotaxin
LPA: Lysophosphatidic acid
LPC: Lysophosphatidylcholine
GPCRs: G protein-coupled receptors
ENTPD: Ectonucleoside triphosphate diphosphohydrolase
GPI: Glycosylphosphatidylinositol
eATP: Extracellular ATP
IDO: Indoleamine 2.3-dioxygenase

## References

[1] Vyas JM (2012). The dendritic cell: the general of the army. Virulence.

[2] Merad M, Manz MG (2009). Dendritic cell homeostasis. Blood.

[3] Steinman RM (2006). Linking innate to adaptive immunity through dendritic cells. Novartis Found Symp.

[4] Sabado RL, Balan S, Bhardwaj N (2017). Dendritic cell-based immunotherapy. Cell Res.

[5] Bol KF, Schreibelt G, Gerritsen WR, de Vries IJ, Figdor CG (2016). Dendritic Cell-Based Immunotherapy: State of the Art and Beyond. Clin Cancer Res.

[6] Palucka K, Banchereau J (2012). Cancer immunotherapy via dendritic cells. Nat Rev Cancer.

[7] Palucka K, Banchereau J (2013). Dendritic-cell-based therapeutic cancer vaccines. Immunity.

[8] Guermonprez P, Valladeau J, Zitvogel L, Thery C, Amigorena S (2002). Antigen presentation and T cell stimulation by dendritic cells. Annu Rev Immunol.

[9] Garg AD, Coulie PG, Van den Eynde BJ, Agostinis P (2017). Integrating next-generation dendritic cell vaccines into the current cancer immunotherapy landscape. Trends Immunol.

[10] Gabrilovich D (2004). Mechanisms and functional significance of tumour-induced dendritic-cell defects. Nat Rev Immunol.

[11] Vicari AP, Caux C, Trinchieri G (2002). Tumour escape from immune surveillance through dendritic cell inactivation. Semin Cancer Biol.

[12] Morehead LC, Cannon MJ (2018). Further clinical advancement of dendritic cell vaccination against ovarian cancer. Ann Res Hosp.

[13] Royer PJ, Tanguy-Royer S, Ebstein F, Sapede C, Simon T, Barbieux I (2006). Culture medium and protein supplementation in the generation and maturation of dendritic cells. Scand J Immunol.

[14] Cintolo JA, Datta J, Mathew SJ, Czerniecki BJ (2012). Dendritic cell-based vaccines: barriers and opportunities. Future Oncol.

[15] Jonuleit H, Schmitt E, Schuler G, Knop J, Enk AH (2000). Induction of interleukin 10-producing, nonproliferating CD4(+) T cells with regulatory properties by repetitive stimulation with allogeneic immature human dendritic cells. J Exp Med.

[16] Dhodapkar MV, Steinman RM, Krasovsky J, Munz C, Bhardwaj N (2001). Antigen-specific inhibition of effector T cell function in humans after injection of immature dendritic cells. J Exp Med.

[17] Schreibelt G, Tel J, Sliepen KH, Benitez-Ribas D, Figdor CG, Adema GJ (2010). Toll-like receptor expression and function in human dendritic cell subsets: implications for dendritic cell-based anti-cancer immunotherapy. Cancer Immunol Immunother.

[18] Saxena M, Bhardwaj N (2018). Re-emergence of dendritic cell vaccines for cancer treatment. Trends Cancer.

[19] Bennaceur K, Chapman J, Brikci-Nigassa L, Sanhadji K, Touraine JL, Portoukalian J (2008). Dendritic cells dysfunction in tumour environment. Cancer Lett.

[20] Schito L, Semenza GL (2016). Hypoxia-inducible factors: master regulators of cancer progression. Trends Cancer.

[21] Huang D, Li C, Zhang H (2014). Hypoxia and cancer cell metabolism. Acta Biochim Biophys Sin (Shanghai).

[22] Avgoustiniatos ES, Scott 3rd WE, Suszynski TM, Schuurman HJ, Nelson RA, Rozak PR (2012). Supplements in human islet culture: human serum albumin is inferior to fetal bovine serum. Cell Transplant.

[23] De Castro M, Orive G, Gascon AR, Hernandez RM, Pedraz JL (2006). Evaluation of human serum albumin as a substitute of foetal bovine serum for cell culture. Int J Pharm.

[24] Krawczyk P, Wojas K, Milanowski J, Rolinski J (2007). The influence of different culture microenvironments on the generation of dendritic cells from non-small-cell lung cancer patients. Arch Immunol Ther Exp (Warsz).

[25] Eljaafari A, Duperrier K, Mazet S, Bardin C, Bernaud J, Durand B (1998). Generation of stable monocyte-derived dendritic cells in the presence of high concentrations of homologous or autologous serum: influence of extra-cellular pH. Hum Immunol.

[26] Pietschmann P, Stockl J, Draxler S, Majdic O, Knapp W (2000). Functional and phenotypic characteristics of dendritic cells generated in human plasma supplemented medium. Scand J Immunol.

[27] Kacani L, Wurm M, Schennach H, Braun I, Andrle J, Sprinzl GM (2003). Immunosuppressive effects of soluble factors secreted by head and neck squamous cell carcinoma on dendritic cells and T lymphocytes. Oral Oncol.

[28] Tang M, Diao J, Cattral MS (2017). Molecular mechanisms involved in dendritic cell dysfunction in cancer. Cell Mol Life Sci.

[29] Onishi H, Morisaki T, Baba E, Kuga H, Kuroki H, Matsumoto K (2002). Dysfunctional and short-lived subsets in monocyte-derived dendritic cells from patients with advanced cancer. Clin Immunol.

[30] Pinzon-Charry A, Maxwell T, Lopez JA (2005). Dendritic cell dysfunction in cancer: a mechanism for immunosuppression. Immunol Cell Biol.

[31] Herber DL, Cao W, Nefedova Y, Novitskiy SV, Nagaraj S, Tyurin VA (2010). Lipid accumulation and dendritic cell dysfunction in cancer. Nat Med.

[32] Cubillos-Ruiz JR, Silberman PC, Rutkowski MR, Chopra S, Perales-Puchalt A, Song M (2015). ER stress sensor XBP1 controls anti-tumor immunity by disrupting dendritic cell homeostasis. Cell.

[33] Cubillos-Ruiz JR, Glimcher LH (2016). Targeting abnormal ER stress responses in tumors: A new approach to cancer immunotherapy. Oncoimmunology.

[34] Mishra A (2017). Metabolic plasticity in dendritic cell responses: implications in allergic asthma. J Immunol Res.

[35] Liu S, Murph M, Panupinthu N, Mills GB (2009). ATX-LPA receptor axis in inflammation and cancer. Cell Cycle.

[36] Chan LC, Peters W, Xu Y, Chun J, Farese Jr RV, Cases S (2007). LPA3 receptor mediates chemotaxis of immature murine dendritic cells to unsaturated lysophosphatidic acid (LPA). J Leukoc Biol.

[37] Bastid J, Cottalorda-Regairaz A, Alberici G, Bonnefoy N, Eliaou JF, Bensussan A (2013). ENTPD1/CD39 is a promising therapeutic target in oncology. Oncogene.

[38] Bono MR, Fernandez D, Flores-Santibanez F, Rosemblatt M, Sauma D (2015). CD73 and CD39 ectonucleotidases in T cell differentiation: beyond immunosuppression. FEBS lett.

[39] Montalban Del Barrio I, Penski C, Schlahsa L, Stein RG, Diessner J, Wockel A (2016). Adenosine-generating ovarian cancer cells attract myeloid cells which differentiate into adenosine-generating tumor associated macrophages - a self-amplifying, CD39- and CD73-dependent mechanism for tumor immune escape. J Immunother Cancer.

[40] Clayton A, Al-Taei S, Webber J, Mason MD, Tabi Z (2011). Cancer exosomes express CD39 and CD73, which suppress T cells through adenosine production. J Immunol.

[41] Maliszewski CR, Delespesse GJ, Schoenborn MA, Armitage RJ, Fanslow WC, Nakajima T (1994). The CD39 lymphoid cell activation antigen. Molecular cloning and structural characterization. J Immunol.

[42] Chadwick BP, Frischauf AM (1997). Cloning and mapping of a human and mouse gene with homology to ecto-ATPase genes. Mamm Genome.

[43] Grinthal A, Guidotti G (2006). CD39, NTPDase 1, is attached to the plasma membrane by two transmembrane domains. Why?. Purinergic signal.

[44] Zhong X, Malhotra R, Woodruff R, Guidotti G (2001). Mammalian plasma membrane ecto-nucleoside triphosphate diphosphohydrolase 1, CD39, is not active intracellularly. The N-glycosylation state of CD39 correlates with surface activity and localization. J Biol Chem.

[45] Airas L, Niemela J, Salmi M, Puurunen T, Smith DJ, Jalkanen S (1997). Differential regulation and function of CD73, a glycosyl-phosphatidylinositol-linked 70-kD adhesion molecule, on lymphocytes and endothelial cells. J Cell Biol.

[46] Mandapathil M, Boduc M, Roessler M, Guldner C, Walliczek-Dworschak U, Mandic R (2018). Ectonucleotidase CD39 expression in regional metastases in head and neck cancer. Acta otolaryngol.

[47] Zhang B, Cheng B, Li FS, Ding JH, Feng YY, Zhuo GZ (2015). High expression of CD39/ENTPD1 in malignant epithelial cells of human rectal adenocarcinoma. Tumour Biol.

[48] Zhang H, Vijayan D, Li XY, Robson SC, Geetha N, Teng MWL (2019). The role of NK cells and CD39 in the immunological control of tumor metastases. Oncoimmunology.

[49] Vijayan D, Barkauskas DS, Stannard K, Sult E, Buonpane R, Takeda K (2017). Selective activation of anti-CD73 mechanisms in control of primary tumors and metastases. Oncoimmunology.

[50] Mandapathil M, Boduc M, Netzer C, Guldner C, Roessler M, Wallicek-Dworschak U (2018). CD73 expression in lymph node metastases in patients with head and neck cancer. Acta otolaryngol.

[51] Gao ZW, Wang HP, Lin F, Wang X, Long M, Zhang HZ (2017). CD73 promotes proliferation and migration of human cervical cancer cells independent of its enzyme activity. BMC cancer.

[52] Ma XL, Shen MN, Hu B, Wang BL, Yang WJ, Lv LH (2019). CD73 promotes hepatocellular carcinoma progression and metastasis via activating PI3K/AKT signaling by inducing Rap1-mediated membrane localization of P110beta and predicts poor prognosis. J Hematol Oncol.

[53] Pellegatti P, Raffaghello L, Bianchi G, Piccardi F, Pistoia V, Di Virgilio F (2008). Increased level of extracellular ATP at tumor sites: *in vivo* imaging with plasma membrane luciferase. PloS one.

[54] Aymeric L, Apetoh L, Ghiringhelli F, Tesniere A, Martins I, Kroemer G (2010). Tumor cell death and ATP release prime dendritic cells and efficient anticancer immunity. Cancer Res.

[55] Coutinho-Silva R, Stahl L, Cheung KK, de Campos NE, de Oliveira Souza C, Ojcius DM (2005). P2X and P2Y purinergic receptors on human intestinal epithelial carcinoma cells: effects of extracellular nucleotides on apoptosis and cell proliferation. Am J Physiol Gastrointest Liver Physiol.

[56] Kroemer G, Galluzzi L, Kepp O, Zitvogel L (2013). Immunogenic cell death in cancer therapy. Annu Rev Immunol.

[57] Antonioli L, Blandizzi C, Pacher P, Hasko G (2013). Immunity, inflammation and cancer: a leading role for adenosine. Nat Rev Cancer.

[58] Yao Y, Levings MK, Steiner TS (2012). ATP conditions intestinal epithelial cells to an inflammatory state that promotes components of DC maturation. Eur J Immunol.

[59] Schnurr M, Toy T, Stoitzner P, Cameron P, Shin A, Beecroft T (2003). ATP gradients inhibit the migratory capacity of specific human dendritic cell types: implications for P2Y11 receptor signaling. Blood.

[60] Novitskiy SV, Ryzhov S, Zaynagetdinov R, Goldstein AE, Huang Y, Tikhomirov OY (2008). Adenosine receptors in regulation of dendritic cell differentiation and function. Blood.

[61] Arab S, Kheshtchin N, Ajami M, Ashurpoor M, Safvati A, Namdar A (2017). Increased efficacy of a dendritic cell-based therapeutic cancer vaccine with adenosine receptor antagonist and CD73 inhibitor. Tumour Biol.

[62] Cekic C, Sag D, Li Y, Theodorescu D, Strieter RM, Linden J (2012). Adenosine A2B receptor blockade slows growth of bladder and breast tumors. J Immunol.

